# Differences in Paediatric intensive care mortality by biological sex: a study of English PICUs using linked administrative datasets

**DOI:** 10.1007/s44253-026-00130-8

**Published:** 2026-07-25

**Authors:** Ofran Almossawi, Bianca L. De Stavola, Katie Harron, Richard G. Feltbower, Joseph Ward, Simon Nadel

**Affiliations:** 1 UCL Great Ormond Street Institute of Child Health, London, WC1N 1EH UK; 2 Great Ormond Street Hospital, London, UK; 3 Child Health Outcomes Research at Leeds (CHORAL), School of Medicine, University of Leeds, Leeds, UK; 4 Kings College Hospital, London, UK; 5 Imperial College Healthcare NHS Trust and Imperial College London, London, UK

**Keywords:** Sex differences, Paediatric intensive care, Mortality, Data linkage, Causal inference, Causal machine learning

## Abstract

**Objective:**

To determine if there is a causal relationship between sex and mortality in Paediatric Intensive Care Units (PICUs).

**Methods:**

A multi-centre, population-based cohort study of English PICUs, designed using the potential outcomes framework and doubly robust methods. Participants were children aged 0 to 8 years with at least one admission to PICU between January 2010 and December 2019, with linked PICU and hospital records. The exposure was biological sex, and the main outcome was the causal difference in mortality between females in PICU and their counterfactual mortality if these females were hypothetically male (Average Treatment effect in the Treated; ATT).

**Results:**

There were 48,500 children, of whom 20,412 (42.1%) were females and 28,088 (57.9%) were males and with a median age at first PICU admission of 0.5 (IQR 0.08–2.02) years. During the first admission, 1,760 (3.63%) children died; of whom, 777/20,412 (3.81%) were female and 983/28,088 (3.50%) were male. The doubly robust estimated causal mortality risk difference captured by the ATT (i.e. excess mortality for girls in PICU), was 0.31% (95% CI 0.01–0.61).

**Conclusions:**

Although boys have a higher rate of death than girls by age in the general population, this mortality trend is reversed in PICU. Evidence of this increased female mortality persists after applying doubly robust causal methods, indicating a causal link. The supposition that all pre-pubertal children have the same responses to critical illness and their management should be re-examined.

**Supplementary Information:**

The online version contains supplementary material available at 10.1007/s44253-026-00130-8.

## Introduction

Mortality in children and young people (CYP) is higher in males than females, with female/male mortality ratios from 2010 to 2019 for England and Wales ranging between 0.7 and 0.8 for ages 0 to 14 years [1, 2]. However, there is some evidence this pattern may be reversed in those admitted to Paediatric Intensive Care Units (PICU), where around 16.4% of CYP deaths occur [3]. Understanding whether there are biological sex-related differences in mortality may be useful for understanding differences in response to disease or treatment, to inform risk stratification on PICU admission, and contribute to efforts to improve survival.

Prior work exploring mortality differences by sex in children admitted to PICU have been limited by small sample sizes and time periods [4, 5], restriction by age [6], or have used methods that are vulnerable to selection bias and residual confounding.

In this study we have used a national dataset of PICU admissions in England to examine if female sex was associated with higher mortality risk in children aged zero to eight years. We used causal inference methods to address concerns related to selection bias and residual confounding arising from the use of observational data.

We set out to establish whether there was a difference in mortality in children admitted to English PICUs between males and females, by adopting a formal causal inference approach [7] using whole-population clinical audit and administrative data.

### Ethics review and approval

Approvals were sought from the NHS Research Authority. Ethics approval was obtained from the London - City & East Research Ethics Committee (REC reference number 19/LO/1396). For the use of data without individual consent, approval under Section 251 of the Confidentiality Advisory Group was granted prior to the start of the project (19CAG0164).

This work was conducted in accordance with International Council for Harmonisation guidelines, which enforces the Declaration of Helsinki, and a protocol approved by the national ethics committee.

### Design and methods

Inclusions: All admissions to English PICUs between 01 January 2010 and 31 December 2019. The date range was to cover a period of at least 10 years to account for any variations in PICU care. The 2010 start date was to account for a change in the data collection method in Paediatric Intensive Care Audit Network (PICANet).

Data from four sources were requested and linked together by NHS Digital (now NHS England):

1. The Paediatric Intensive Care Audit Network (PICANet) [8]
2. Hospital Episode Statistics (HES) for children
3. Hospital Episode Statistics (HES) for mothers
4. Office for National Statistics (ONS) mortality records

PICANet is a UK national audit dataset used for research and for national benchmarking of PICU outcomes. HES is the data collected for all admissions in England [9] primarily for the purposes of financial reimbursement, but contains information on treatments, outcomes, diagnoses, demographics [9] (among other information) which can be used for research. The ONS mortality record are the civil registrations of deaths which are linked to HES [10].

The data linkage process is described in the Appendix, Section  1, Figure S1. These datasets captured pre-PICU admission factors from both mothers and infants that could impact on differential mortality experienced by girls and boys admitted to PICU (both during PICU admission and post PICU discharge). HES data comprised Admitted Patient Care (APC) records on inpatient and day case admissions, which include detailed information on diagnoses (coded using ICD10 diagnosis codes) and patient characteristics (e.g. ethnicity, age and area-level deprivation), also important for controlling sex differentials in mortality.

Data on mortality was gathered from two sources: the PICANet dataset for deaths in PICU, and linked ONS mortality records for deaths after discharge from PICU.

### Inclusion and exclusion criteria

All children aged zero to eight years captured within the PICANet dataset between January 2010 and December 2019 were included if they had a linked HES record. The upper age limit of eight years was a result of the availability of linkage to maternal hospital records. Our cohort corresponds to children born in NHS-funded hospitals between April 2002 and December 2019. Children were linked to their maternal HES record using a mother-baby linkage algorithm described elsewhere [11]. We restricted our analysis to the first PICU admission for each child, and did not examine multiple PICU admissions. Children who were not born in England, those born outside NHS institutions (and therefore not captured in HES), and those who could not be linked to their maternal record were excluded (Appendix Section  1, Figure S1).

## Main outcome measures

The primary outcome was all-cause mortality in PICU. A secondary outcome was mortality up to six years after discharge from PICU.

### Covariates

**Fig. 1:**
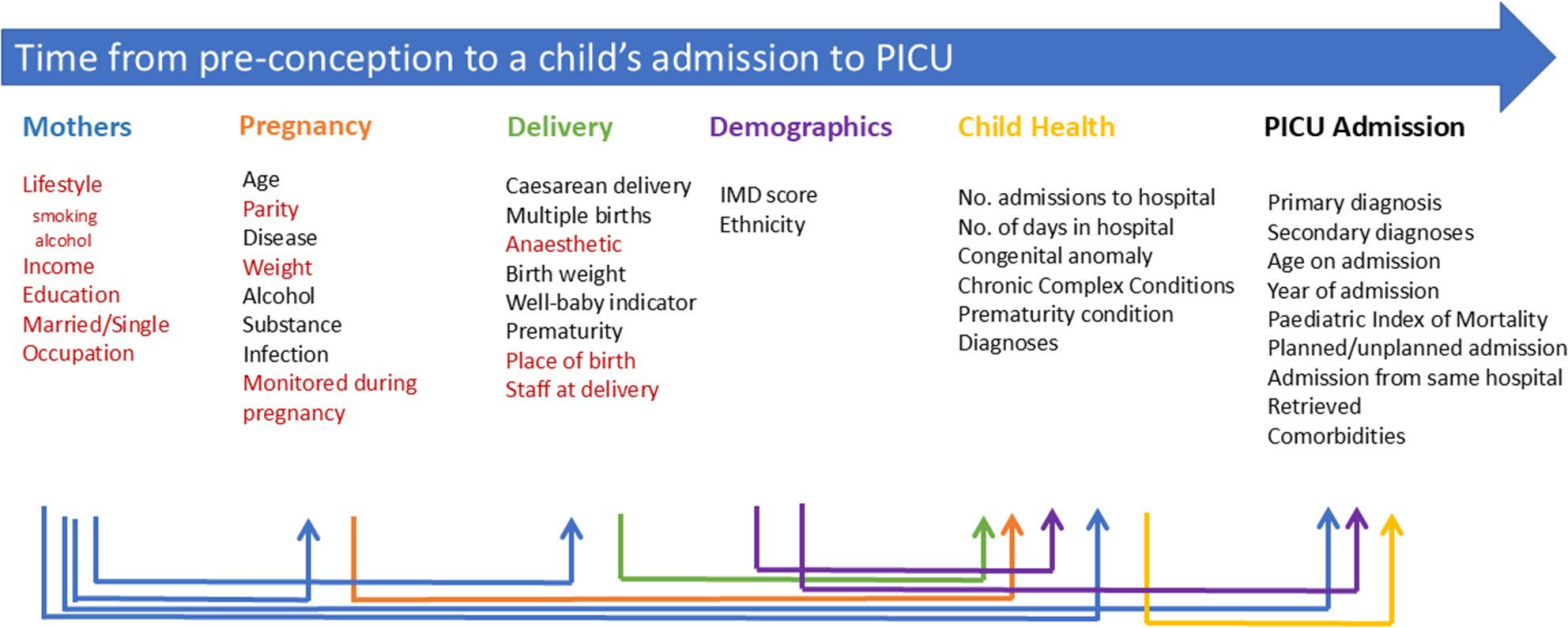
Hypothesised relationships between maternal and child characteristics, biological sex, and death in PICU. The characteristics in red were not available. Coloured arrows denote the direction of relationships between variables (variables to the left are causes of covariates to the right). Disease in pregnancy was any diagnosis during pregnancy. This diagram illustrates the temporality of the characteristics relative to each other and is not meant to be an exhaustive list

We captured the following covariates in our data (Fig. 1) which could be associated either with the primary outcome or the exposure sex: maternal characteristics during pregnancy and delivery (age, pregnancy or delivery-related diagnoses [defined using ICD10 codes, Appendix Section  2], mode of delivery [C-section or not], multiple birth, birth weight, whether the baby had a Well-baby Indicator at birth, preterm birth [< 37 weeks of gestation]); child health-related variables (number of hospital admissions and total number of inpatient days in the year prior to PICU admission, chronic complex conditions [defined using ICD10 codes, Appendix Section  2]); PICU-related variables (diagnosis at PICU admission, secondary diagnoses, age on admission, year of admission, Paediatric Index of Mortality [12] [PIM, as an indicator of severity of illness on admission to PICU]; planned or unplanned PICU admission, whether the child was admitted to PICU from the same hospital or admitted to PICU by a retrieval service); Socioeconomic position using deciles of the Index of Multiple Deprivation (IMD) score in HES based on residential postcode [13]; and ethnicity.

### Statistical methods

#### Descriptive analysis

We compared our study cohort to the whole PICANet population (i.e., including those who were not linked to HES), to assess representativeness of our cohort and to describe its characteristics according to sex, ethnicity, primary diagnostic group, year of admission to PICU, PIM score, and length of stay in PICU.

As an additional comparison, we used data on mortality rates in the general population of children in the UK between 2010 and 2020, obtained from the ONS child mortality tables for England and Wales [14].

To compare male and female mortality by severity of disease on admission to PICU, as captured by the PIM score (PIM version 3 used in this study, we refer to it as PIM throughout), we divided the mortality risk (derived from PIM) into deciles and calculated the mortality risks for patients within these deciles.

#### Causal reasoning

Our causal analysis was conducted following the STRATOS guidelines [15]. As we wished to assess whether there was a sex difference in mortality in children in English PICUs, the causal contrast we could identify from our data was the difference between the observed mortality rate in PICU for females and their mortality rate in PICU if they had been males (the counterfactual). This is known as the Average Treatment effect in the Treated (ATT) and we expressed it as a risk difference (i.e., difference in mortality risk, or excess mortality for females) as this was the most useful scale for public health evaluation. For completeness we also report this comparison on the risk ratio scale.

We used directed acyclic graphs (DAGs) to represent our assumptions regarding the relationships among all the variables of interest (sex, death in PICU, socio-demographic and clinical variables, Appendix Section  3, Figure S2). This enabled us to determine which variables to include in our analysis to control for the likely confounding and selection bias that would affect crude comparisons of mortality rates between sexes. We assumed that the large number of covariates included in these analyses would be expected to capture the impact of most/all the confounders affecting any observed sex-PICU mortality association.

#### Analysis methods

As a naïve non-causal measure, we calculated the percentage of males and females who died in PICU and calculated the crude risk ratio and risk difference for female versus male mortality.

For our causal analysis, we first used g-computation (standardisation) using logistic regression to estimate the ATT. We also used two doubly robust methods of estimation. The first consists of estimating the probability of a child in PICU being either male or female, given the set of identified confounders (the propensity score) and then using these propensity scores to estimate the ATT within an Augmented Inverse Probability Weighting [16] (AIPW) approach, assuming that the propensity score reweighting had resulted in groups that were comparable at PICU admission. For the second method we used Targeted Maximum Likelihood Estimation [17] (TMLE) in which we incorporated an ensemble of machine learning algorithms to improve the specification of the propensity score before including it into the estimation algorithm (Appendix, Section  4). These three methods of causal analysis estimation are subject to a number of assumptions (Appendix, Section  3).

Our main analysis was conducted using only records with complete information on all covariates. As a sensitivity analysis, we re-estimated the g-computation (using a logistic regression model), and AIPW-based ATT using a multiple imputation approach to deal with missing values (48500/64495, 75% complete records) (Appendix, Section  5, and Table S1). For all analyses except for AIPW and TMLE, the 95% confidence intervals were bootstrapped, with resamples stratified by the outcome.

For the secondary outcome, mortality in the six years after discharge from PICU, we compared the survival probabilities from the point of admission to PICU and up to six years after that, separately by sex, using the non-parametric Kaplan-Meier method. The six-year cut off was based on availability of death records.

## Results

48,500 children admitted to PICU between 1st January 2010 and 31st December 2019, aged 0–8 years and linked to their HES and maternal HES records were included (Table 1). Among them, 58% (*n* = 28,088) were males and 42% (*n* = 20,412) were females. Children in the analysis cohort were representative of all children in PICUs during the study period in terms of their main characteristics (Appendix Section  6, Table S2).

Across the ten-year study period, more boys than girls were admitted to PICU (Table 2). Using population estimates as a denominator, the ratio of boys to girls who were admitted to PICU ranged from 1.18 to 1.26.

Males and females in our study had almost identical PIM scores, suggesting no difference in severity of illness and risk of death on admission to PICU. After stratifying by risk of death (calculated from PIM score) deciles, we observed identical PIM-derived risk of death within each decile, however, the observed crude mortality risk was higher for girls than boys (Appendix, Section  7, Table S3).

Of the 48,500 children included, 1,760 died; 777/20,412 (3.81%) were females and 983/28,088 (3.50%) males. The crude risk ratio for mortality of females compared to males was 1.10 (95% CI 0.99 to 1.20), and the crude risk difference in PICU mortality rates was 0.31% (95% CI -0.02 to 0.65).

**Fig. 2:**
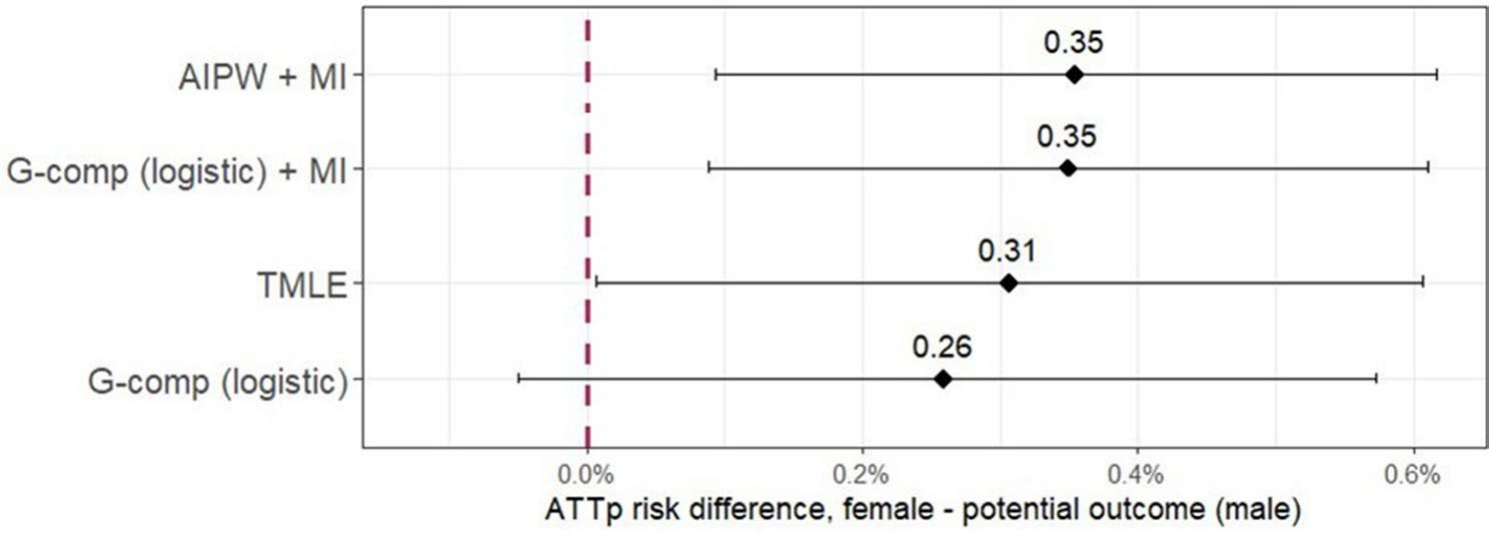
Summary of the methods used for estimating the $\:AT{T}_{P}$. $\:AT{T}_{P}$: PICU average causal effect in the exposed (females), AIPW: Augmented inverse probability weights, G-comp(logistic): g-computation based on a logistic regression model; TMLE: Targeted maximum likelihood estimate. The horizontal lines are 95% confidence intervals. The 𝐴𝑇𝑇_𝑃_ is the causal contrast which is the risk of death (expressed as a percentage %) in females minus the potential outcome of females, had they been males, among the female PICU population

**Fig. 3:**
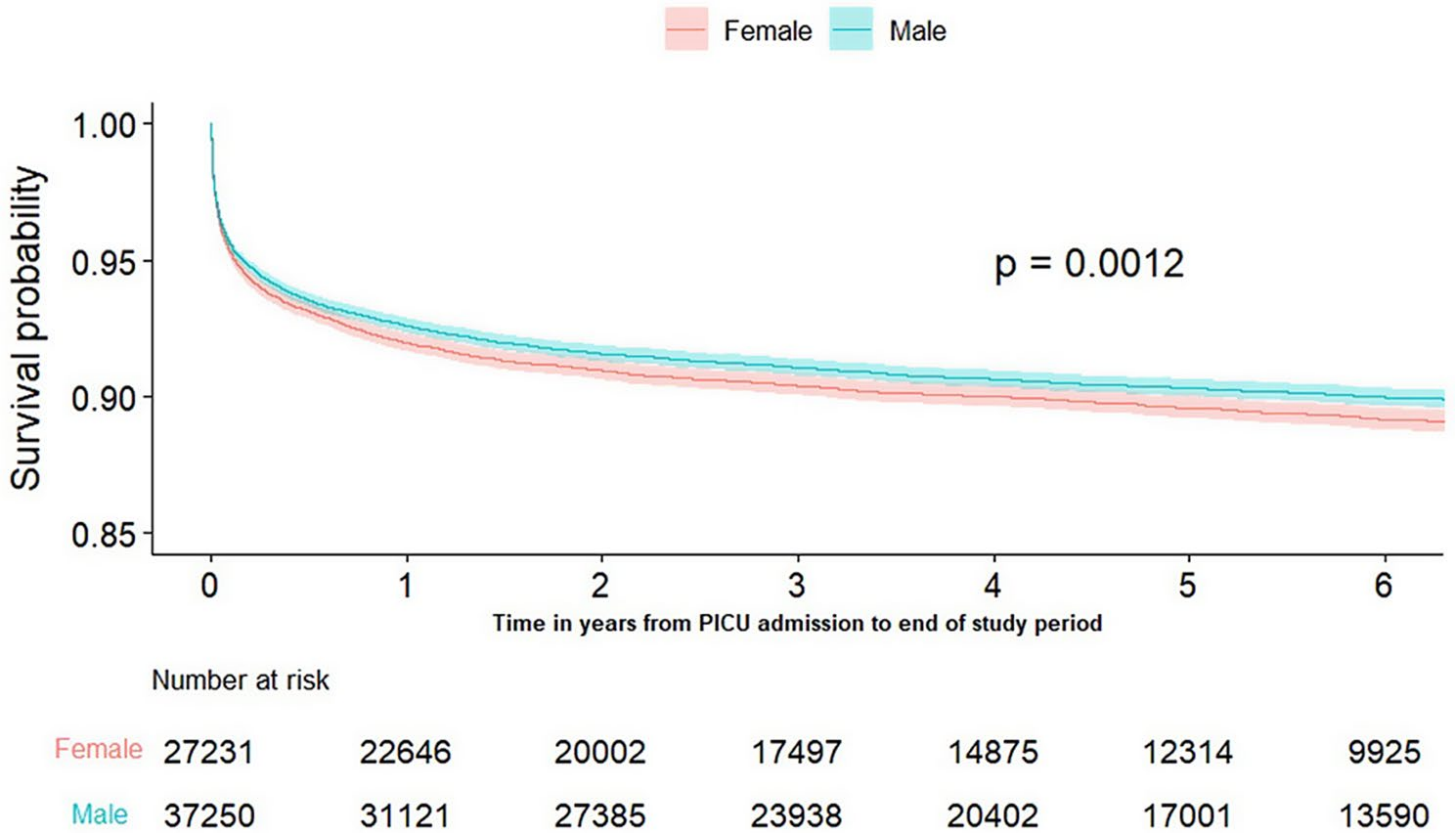
Survivor functions for males and females ages 0 to 8 years, from first PICU admission date to any death or censoring for the analysis dataset during any PICU admission between 2010–2019. The outcome of death was censored at 6 years. The p-value for the log-rank test for the difference in survival curves is given within the figure. This plot is based on all the complete cases, *N* = 64,495

The estimated ATT, using the most robust estimator which is TMLE, and expressed as a risk difference was 0.31% (95% CI 0.01–0.61). This represents a comparison of the observed risk in females to the risk that would have been found had all females been males. Both the crude and the causal estimates indicate a higher risk of death in females versus males (Fig. 2), even after accounting for all the identified confounders. The multiple imputation results were consistent with the complete records analysis (Fig. 2). These differences also persisted in our secondary descriptive analysis evaluating post-PICU survival up to 6 years after admission (Fig. 3). These analyses indicate a negative impact of PICU admission on mortality for females, which starts during the PICU admission and persists in the years following discharge from PICU.

**Table 1:** Study population characteristics for the analysis cohort, *N* = 48,500

Characteristic	Female, *N* = 20,412	Male, *N* = 28,088
**Birth weight (grams)**	2,881 (832) 3,020	2,995 (876) 3,150
**Length of stay in PICU (days)**	4.9 (11.5) 2.4	4.7 (10.5) 2.4
**Number of multiple admissions to PICU**	2 (1) 1	2 (1) 1
**Gestation in weeks**	34 (11) 38	34 (11) 38
**PICU admission age in months**	19 (26) 6	18 (25) 5
**PIM (risk of death)**	0.04 (0.09) 0.02	0.04 (0.09) 0.02
**Maternal age in years**	29 (6) 29	29 (6) 29
**Number of maternal diagnoses**	10 (11) 7	10 (11) 6
**Primary diagnostic group in PICU**		
Cardiovascular	6622 (32.4%)	8289 (29.5%)
Endocrine / metabolic	527 (2.6%)	608 (2.2%)
Gastrointestinal	1229 (6.0%)	1879 (6.7%)
Infection	1216 (6.0%)	1688 (6.0%)
Musculoskeletal	299 (1.5%)	434 (1.5%)
Neurological	2253 (11.0%)	2975 (10.6%)
Oncology	635 (3.1%)	773 (2.8%)
Other	1305 (6.4%)	2020 (7.2%)
Respiratory	5979 (29.3%)	8760 (31.2%)
Trauma	347 (1.7%)	662 (2.4%)
**Other diagnoses in PICU**,** categorised**		
0	10,773 (52.8%)	15,005 (53.4%)
1	5053 (24.8%)	6923 (24.6%)
2	2373 (11.6%)	3085 (11.0%)
3	2213 (10.8%)	3075 (10.9%)
**Planned versus unplanned admission to PICU**		
Planned	7323 (35.9%)	9340 (33.3%)
Unplanned	13,089 (64.1%)	18,748 (66.7%)
**IMD Decile**		
0*	103 (0.5%)	125 (0.4%)
1	3701 (18.1%)	4980 (17.7%)
2	2937 (14.4%)	4001 (14.2%)
3	2434 (11.9%)	3523 (12.5%)
4	2113 (10.4%)	2885 (10.3%)
5	1845 (9.0%)	2429 (8.6%)
6	1668 (8.2%)	2383 (8.5%)
7	1515 (7.4%)	2092 (7.4%)
8	1476 (7.2%)	2054 (7.3%)
9	1317 (6.5%)	1865 (6.6%)
10	1303 (6.4%)	1751 (6.2%)
**Ethnicity**		
Asian	2701 (13.2%)	3665 (13.0%)
Black	1215 (6.0%)	1731 (6.2%)
Mixed	913 (4.5%)	1198 (4.3%)
Other	601 (2.9%)	753 (2.7%)
White	14,982 (73.4%)	20,741 (73.8%)
**Indicator of health at birth**		
No	16,717 (81.9%)	23,363 (83.2%)
Yes	3695 (18.1%)	4725 (16.8%)
**Prematurity**,** cutoff at < 37 weeks**		
No	14,323 (70.2%)	19,525 (69.5%)
Yes	6089 (29.8%)	8563 (30.5%)
**Admission to PICU from the same hospital**		
No	7894 (38.7%)	11,010 (39.2%)
Yes	12,518 (61.3%)	17,078 (60.8%)
**Emergency admissions pre-PICU**		
Elective	18,666 (91.4%)	25,614 (91.2%)
Emergency	1746 (8.6%)	2474 (8.8%)
**Born by C-section**		
No	13,557 (66.4%)	18,245 (65.0%)
Yes	6855 (33.6%)	9843 (35.0%)
**Risk factors during pregnancy or delivery**		
No	17,261 (84.6%)	23,832 (84.8%)
Yes	3151 (15.4%)	4256 (15.2%)
**Source of admission to hospital**		
Medical facility	4265 (20.9%)	5954 (21.2%)
Nonmedical facility	16,147 (79.1%)	22,134 (78.8%)
**Year of admission to PICU**		
2010	2083 (10.2%)	2902 (10.3%)
2011	2110 (10.3%)	2906 (10.3%)
2012	2258 (11.1%)	2950 (10.5%)
2013	2112 (10.3%)	2994 (10.7%)
2014	2028 (9.9%)	2874 (10.2%)
2015	2120 (10.4%)	2838 (10.1%)
2016	2144 (10.5%)	2813 (10.0%)
2017	1940 (9.5%)	2685 (9.6%)
2018	1892 (9.3%)	2656 (9.5%)
2019	1725 (8.5%)	2470 (8.8%)
**Maternal multiple births**		
No	19,352 (94.8%)	26,705 (95.1%)
Yes	1060 (5.2%)	1383 (4.9%)
**Recorded comorbidities in PICU**,** categorised**		
No comorbidities	14,450 (70.8%)	20,205 (71.9%)
1 comorbidity	3095 (15.2%)	4084 (14.5%)
2 comorbidities	1313 (6.4%)	1696 (6.0%)
3 comorbidities	1554 (7.6%)	2103 (7.5%)
**Risk factors in pregnancy**		
No	17,429 (85.4%)	24,038 (85.6%)
Yes	2983 (14.6%)	4050 (14.4%)
**Risk factors during delivery**		
No	20,313 (99.5%)	27,969 (99.6%)
Yes	99 (0.5%)	119 (0.4%)
**Chronic complex conditions**		
No	11,793 (57.8%)	16,413 (58.4%)
Yes	8619 (42.2%)	11,675 (41.6%)
**Perinatal infection**		
No	16,099 (78.9%)	21,676 (77.2%)
Yes	4313 (21.1%)	6412 (22.8%)
**Congenital anomaly**		
No	7479 (36.6%)	10,080 (35.9%)
Yes	12,933 (63.4%)	18,008 (64.1%)
**Maternal substance misuse**		
No	20,294 (99.4%)	27,923 (99.4%)
Yes	118 (0.6%)	165 (0.6%)
**Prematurity condition**		
No	15,693 (76.9%)	20,808 (74.1%)
Yes	4719 (23.1%)	7280 (25.9%)

Continuous variables: mean (SD) median; Categorical variables: n (%)

*IMD Decile = 0 are children who have a birth record in England and an PICU admission record in England, but are resident in Wales during the study period

**Table 2:** Number and year-specific percentage of PICU admissions by sex and percentage of the year-specific general population of England admitted to PICU

Year admitted	Admitted to PICU					% of population in England admitted to PICU	
	Female	Male	Total	% Female	% Male	Female	Male
2010	7,169	9,321	16,490	43.47	56.53		
2011	6,893	9,165	16,058	42.93	57.07	0.08	0.10
2012	7,398	9,345	16,743	44.19	55.81	0.09	0.10
2013	7,271	9,685	16,956	42.88	57.12	0.08	0.11
2014	7,174	9,529	16,703	42.95	57.05	0.08	0.11
2015	7,357	9,636	16,993	43.29	56.71	0.09	0.11
2016	7,509	9,692	17,201	43.65	56.35	0.09	0.11
2017	7,302	9,558	16,860	43.31	56.69	0.09	0.11
2018	7,474	9,957	17,431	42.88	57.12	0.09	0.12
2019	7,656	10,018	17,674	43.32	56.68	0.10	0.13
Total	73,203	95,906	169,109	43.29	56.71		

## Discussion

This study is the first to use robust causal methods to address differences in mortality rates between males and females in PICUs. We found female sex to be associated with higher mortality, which was not explained by sociodemographic or clinical risk factors. This finding demonstrates that the higher mortality observed amongst male children in the general population is reversed in those admitted to a PICU.

Since mortality is a rare outcome in PICU, this increase in risk of death will only be seen on a population level. We estimate that for every 1000 female admissions to PICU there are more than 3 extra deaths compared to males with the same mortality risk score and baseline characteristics. For a typical PICU with around 500 admissions a year, there would be one to two additional female deaths per year compared with males with the same severity of illness.

### Comparison with previous literature

Two small scale analyses of infants with infections admitted to UK PICUs reported higher numbers of male admissions over a two-year period, but a relatively greater female mortality [4, 5]. A larger study of all infants (0 to 12 months old) admitted to PICU over an 11-year period from 2005 to 2015 in the UK^6^ also showed a higher mortality rate for females relative to males, after controlling for prognostic factors known to be unequally distributed between boys and girls. However, results from this study may have been biased: Firstly, the variable selection was not based on any causal reasoning and thus important confounding variables may have not been controlled for, leading to residual confounding; Secondly, there could have been an impact of restricting the analyses to the PICU population which, if unrecognised, could lead to selection bias. Thirdly, the analysis was only conducted on 50% of the whole population due to missingness affecting a number of variables. Finally, age was restricted to < 12 months, which did not allow for variability in cause of death for older ages that could explain sex differences in mortality.

### Strengths and limitations

This is the first national study to address the question of sex-related differences in mortality in English PICUs. We included a large number of patients admitted over a 10 year period. The study design had three key strengths to ensure robustness of the findings: First, we applied a causal approach to address the limitations of observational designs; second, we used data linkage to ensure we have a complete set of characteristics which were identified by the causal study design; finally, we employed different methods to show the consistency of our findings, including two doubly robust methods: Augmented Inverse Probability weighting and Targeted Maximum Likelihood Estimation. For the latter (also known as targeted learning or causal machine learning), we incorporated several machine learning algorithms to improve our model specification and mitigate bias arising from an observational design. Our conclusion is consistent across various statistical approaches.

The main limitation our analysis is subject to is that there is always a risk of unmeasured confounding despite our best efforts to account for all known characteristics.

### Meaning and mechanisms

The exact mechanism for increased mortality in critically ill female children remains unclear, and should be the focus of future studies. A recent study in adult intensive care patients [18] suggested that when a diagnosis is less prevalent in females, they tend to have a worse survival outcome compared to males, which is what we observed in this study. Similarly, when a diagnosis is less prevalent in males, they tend to have worse survival outcomes compared to females (Appendix, Section  7, Table S4 and S5).

Males and females in our study had almost identical PIM scores, suggesting no difference in risk of death on admission to PICU. Our results suggest the method used to assess risk of mortality on admission to PICU may need to be amended, and that including sex as a risk factor for mortality should be considered.

The sex-related differences in mortality trends identified in this study may not solely be attributable to biological differences between males and females. Historically, many therapeutic interventions have been developed and validated in predominantly male populations, and this may influence the applicability of evidence-based treatment strategies across sexes. As a result, differences in outcomes could also reflect variation in treatment responses, treatment practices, or other aspects of clinical care, in addition to differences in underlying disease processes. Although these mechanisms could not be examined within the present study, they warrant further investigation and should be considered when interpreting the observed associations.

## Conclusion

We have provided strong evidence to suggest that female biological sex is associated with higher mortality in PICU compared to male sex with the same severity of illness in children up to eight years of age. This has implications for understanding critical illness in young children, where the presumption has been that pre-pubertal children of either sex have identical pathophysiology and responses to treatment. The mechanism underlying this sex difference in outcome requires further investigation.

## Supplementary Information

Below is the link to the electronic supplementary material.

Supplementary Material 1

## Data Availability

The data used in this study were obtained from NHS England (NHS Digital at the time of application) and the Paediatric Intensive Care Audit Network (PICANet). The data are subject to restrictions through data sharing agreements. Access to the data may be requested through applications to HQIP for the PICANet data and NHS England for the HES data.

## Abbreviations

AIPW: Augmented Inverse Probability Weighting
ATT: Average Treatment Effect in the Treated
DAG: Directed Acyclic Graph
HES: Hospital Episode Statistics
ICD10: International Classification of Diseases version 2010
IMD: Index of Multiple Deprivation
ONS: Office for National Statistics
PICANet: Paediatric Intensive Care Audit Network
PICU: Paediatric Intensive Care
PIM: Paediatric Index of Mortality
TMLE: Targeted Maximum Likelihood Estimation

## References

[1] Office for National Statistics Deaths broken down by age, sex, area and cause of death. https://www.ons.gov.uk/peoplepopulationandcommunity/birthsdeathsandmarriages/deaths#publications

[2] Department of Economic and Social Affairs (2020) Demographic Yearbook. https://unstats.un.org/unsd/demographic-social/products/dyb/dybsets/2020.pdf

[3] Paediatric Intensive Care Audit Network (PICANet) (2024) National Paediatric Critical Care Audit State of the Nation Report 2024. https://www.picanet.org.uk/wp-content/uploads/sites/25/2024/12/PICANet-NPCCA-State-of-the-Nation-Report-2024_v1.0-12Dec2024.pdf

[4] Amer K, Roy RB, Nadel S (2012) Gender mortality differences of infants on PICU? A preliminary analysis. Arch Dis Child 97:A36–A37

[5] Amer K, Nadel S, Basu-Roy R (2013) G57(P) Gender Mortality Differences of Infants on PICU? An Elaboration on Additional Analysis. Arch Dis Child 98:A30–A30

[6] Almossawi O, O’Brien S, Parslow R, Nadel S, Palla L (2021) A study of sex difference in infant mortality in UK pediatric intensive care admissions over an 11-year period. Sci Rep 11:21838 34750426 10.1038/s41598-021-01173-x PMC8575897

[7] Goetghebeur E, Cessie S, le, Stavola BD, Moodie EE, Waernbaum I (2020) Formulating causal questions and principled statistical answers. Stat Med 39 10.1002/sim.8741 PMC7756489 32964526

[8] PICANet – Paediatric Intensive Care Audit Network for the UK and Ireland. https://www.picanet.org.uk/

[9] Data Services, NHS England (NHSE) (2024). NHS England: Hospital Episode Statistics (HES) Admitted Patient Care (APC) Dataset. 10.71760/UKLLC-DATASET-00361-03

[10] Data Services, NHS England (NHSE) (2024) NHS England: Civil Registration of Deaths Dataset - Dated 23 May 2024. 10.71760/UKLLC-DATASET-00368-02

[11] Harron K, Gilbert R, Cromwell D, Meulen J (2016) van der. Linking Data for Mothers and Babies in De-Identified Electronic Health Data. PLoS ONE 11:e0164667 27764135 10.1371/journal.pone.0164667 PMC5072610

[12] Dewi G et al (2020) Pediatric Index of Mortality 3 score as a predictor for the outcomes of critically ill patients. Paediatr Indonesiana 60:328–333

[13] Indices of Multiple Deprivation: how to explore the data in your area - Superhighways. https://superhighways.org.uk/latest/how-to-use-the-indices-of-mult/

[14] Office for National Statistics Child mortality (death cohort) tables in England and Wales. https://www.ons.gov.uk/peoplepopulationandcommunity/birthsdeathsandmarriages/deaths/datasets/childmortalitystatisticschildhoodinfantandperinatalchildhoodinfantandperinatalmortalityinenglandandwales

[15] Stavola BD et al STRATOS TG7: Causal inference

[16] Kurz CF (2022) Augmented Inverse Probability Weighting and the Double Robustness Property. Med Decis Mak 42:156–167 10.1177/0272989X211027181 PMC8793316 34225519

[17] van der Laan MJ, Rubin D (2006) Targeted Maximum Likelihood Learning. Int J Biostatistics 2 10.2202/1557-4679.1211 PMC3126670 21969992

[18] Modra LJ, Higgins AM, Pilcher DV, Bailey MJ, Bellomo R (2022) Sex Differences in Mortality of ICU Patients According to Diagnosis-related Sex Balance. Am J Respir Crit Care Med 206:1353–1360 35849500 10.1164/rccm.202203-0539OC PMC9746862

