# Supplementary material for: Differences in Paediatric intensive care mortality by biological sex: a study of English PICUs using linked administrative datasets
Source: Intensive Care Med Paediatr Neonatal. 2026 Jul 25;4(1):19. doi: 10.1007/s44253-026-00130-8 (PMC13407575; doi:10.1007/s44253-026-00130-8)
Supplement: Supplementary file 1 — Supplementary Material 1 [file 44253_2026_130_MOESM1_ESM.pdf]

Appendix

1. Cohort generation and data flow

Figure S1. Derivation of the study population and final sample size of 48,500 after selecting only the complete records

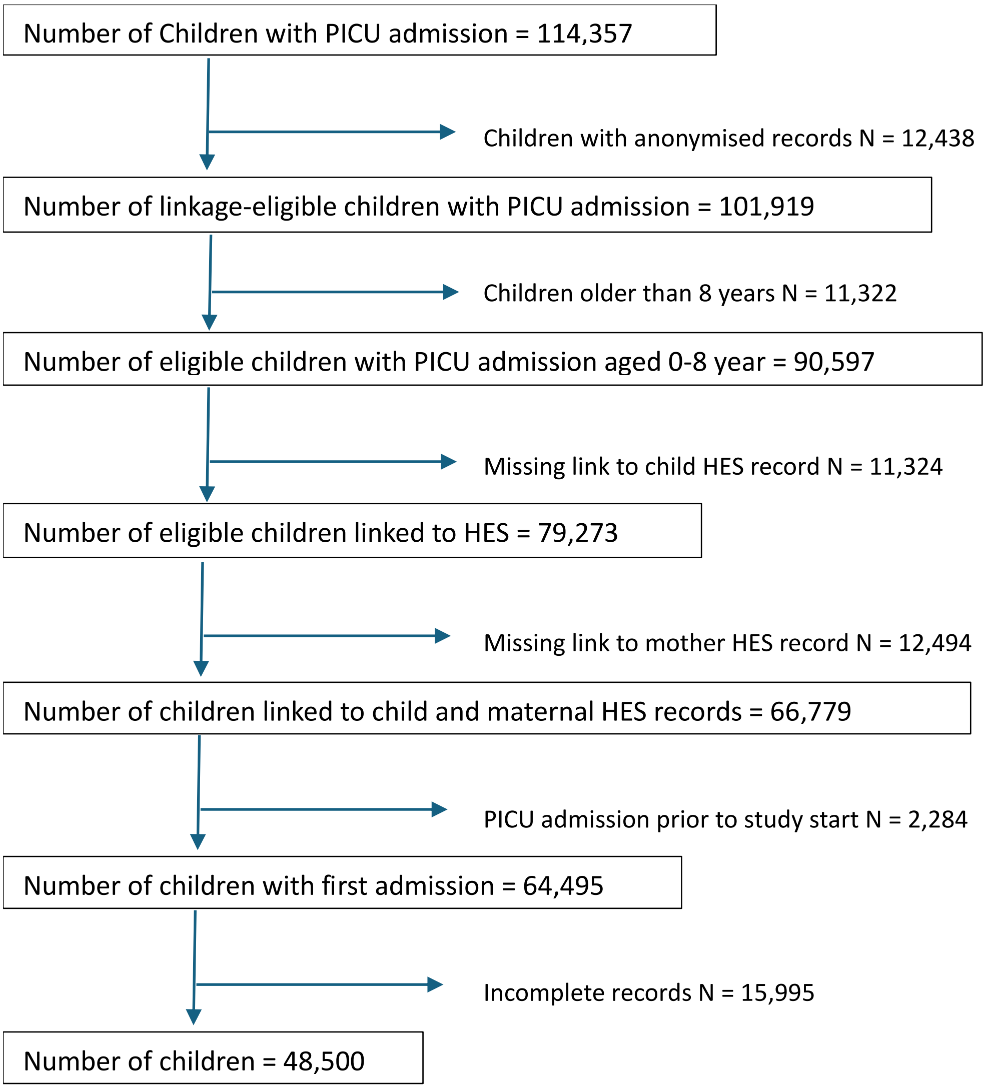

2. Diagnostic codes

List of ICD10 codes for Chronic Complex Conditions and other variables used in the analysis.

Pregnancy risk-factors

Intrauterine fetal death	O364, P95
Eclampsia	O14, O15
Gestational hypertension	O13, O16
Placental abruption or infarction	O45, O431, O438, O439
Uterine rupture	O710, O711
Diabetes in pregnancy	P700, O24, E10-E14

Delivery risk factors

Birth trauma	P10-P15
Complications of delivery	P03
Hypoxia	P20-P21
Amniotic fluid embolism	O881
Chorioamnionitis	O411 P027-P029
Umbilical cord problem	P020, P024-P026
Fetal hemorrhage	P50, P51, P53, P54
Maternal hemorrhage	O430
Umbilical cord problem	O69
Placental-transfusion syndrome	O430

Neonatal medical conditions

Prematurity-related conditions:

Necrotising enterocolitis	P77
Intraventricular hemorrhage	P52, P912
Retinopathy of prematurity	H351
Respiratory distress syndrome	P22

Congenital anomalies:

Q00-Q07, Q10.4, Q10.7, Q11-Q12, Q13.0-Q13.4, Q13.8, Q13.9, Q14-Q16, Q20-Q26, Q18.8, Q30-Q37, Q38.0, Q38.3, Q38.4, Q38.6-Q38.8, Q39, Q40.2, Q40.3, Q40.8, Q40.9, Q41, Q42, Q43.1, Q43.3-Q43.7, Q43.9, Q44, Q45, Q50.0, Q51, Q52.0-Q52.2, Q52.4, Q54.0-Q54.3, Q54.8, Q54.9, Q55.0, Q55.5, Q56, Q60.1, Q60.2, Q60.4-Q60.6, Q61, Q62.0-Q62.6, Q62.8, Q63.0-Q63.2, Q63.8, Q63.9, Q64, Q65.0-Q65.2, Q65.8, Q65.9, Q67.5, Q68.2, Q68.3-Q68.5, Q71-Q73, Q74, Q75.0, Q75.1, Q75.3-Q75.9, Q76.1-Q76.4, Q77, Q78, Q79.0, Q79.2-Q79.5, Q79.6, Q79.8, Q82.0-Q82.4, Q82.9, Q86.2, Q85, Q86.0, Q86.1, Q86.8, Q87.8, Q89.1, Q89.2, Q89.3, Q89.7-Q89.9, Q90-Q93, Q95.2, Q95.3, Q97, Q99

Complex chronic conditions:

B20-B23, D55, D561, D562, D570-D572, D58, D80-D84, D898, D899, E343, E70-E730, E74, E76-E79, E803-E807, E83-E85, E881, E882, E888, E889, F70, F72, F73, F842, G10-G12, G20, G23, G240-G242, G248, G250-G256, G318, G319, G40, G41, G71, G72, G80-G82, G901, G903, G904, G91, G940-G942, G95, G99, I42, I44, I45, I47-I49, I515, K44, K50-K51, K73-K74, K754, K758-K760, M41, N18, P27, P90

Substance-related risk factors:

Neonatal abstinence syndrome	P961
Noxious influences	P04

Perinatal infections:

Perinatal infection	P35-P39
Meningitis or encephalitis	G00-G09

3. Causal reasoning

Assumptions for causal identification of the estimand of interest in this study (ATT):

1. **No interference** This implies that the exposure of one individual does not affect the PO of another. In this setting, being biologically male or female is an assignment at birth which is not expected to influence the PO of other individuals. It is also safe to assume that this assignment will not change throughout the study.
2. **Consistency** Through the assumption of consistency, we are able to link the POs with the data. It states that the PO Y_a is equal to the observed outcome Y if the actual exposure A is equal to a. We therefore say that $Y = Y_a$ if $A = a$, for all levels of A.
3. **Conditional Exchangeability** This is the assumption that the assignment of exposure is independent of the POs, given a set of covariates (which I will denote $\boldsymbol{L}$ for generality). In other words, there is no unmeasured confounding. For this study, exposure assignment (to sex) in the general population is a random process therefore independent of the POs . However, for the children in PICU we assume that this assumption is met conditionally on the minimally sufficient adjustment set of variables.
4. **Positivity** This last assumption states that the assignment of the exposure is not zero for every value of $\boldsymbol{L}$ within PICU

Directed Acyclic Graphs (DAGs):

This study conditions on PICU admission by design since the analysis is restricted to those admitted to PICU (we do not have access to the full population data). The total effect of sex on mortality in the general population when conditioning on PICU cannot be estimated due to conditioning on an intermediate variable or a descendant of an intermediate, in this case PICU admission, which is a consequence of the design.

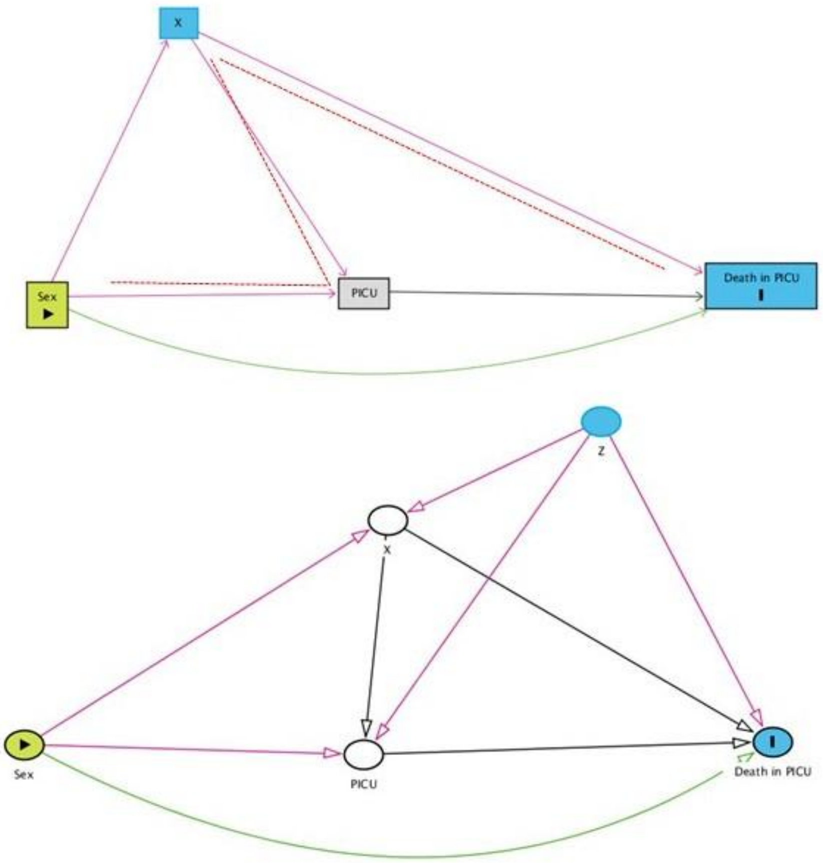

Figure S2. Top DAG: Relationship between sex, PICU admission, and death in PICU. $\boldsymbol{X}$ *is a set of variables (in relation to the causal estmands of* ACE *or* ATT*) that can cause admission to PICU and also have an effect on mortality. The dashed red line represents an induced association between sex and death due to conditioning on PICU that involves the variables* $\boldsymbol{X}$*. The grey box PICU represents conditioning (or adjustment) by PICU admission.* Bottom DAG: *Relationship between sex and mortality in PICU and the biased pathways involving variables from the set* $\boldsymbol{Z}$**.** *Note that now:* $\boldsymbol{X} + \boldsymbol{Z} = \boldsymbol{L}$*, that is the minimum set of confounding variables*

4. Machine learning algorithms

List of learners (machine learning algorithms) included in the ensemble learner and their tuning parameters.

Mean learner

This is a reference for the mean of the dependent outcome to gauge the ML algorithms against.

Generalised linear model

This was a logistic regression for classification of the outcome. It is also the most common model used in the analysis of any PICU outcomes data.

LASSO

A penalised regression which removes any zero coefficients. The hyperparameters were set to lambda = 1, performance measure = deviance, cross validation folds stratified by outcome, and outcome type = binomial. For tuning the lambda parameter, we specified a lambda grid of 100 values, then selected the minimum lambda using 5-fold cross validation.

XG boost

A gradient boosting tree based method which accounts for interactions and non-linear relationships. The hyperparameters were set to: number of rounds = 50, objective = binary logistic, and learning rate = 0.3. The algorithm was tuned using iterations of rounds = 20, 30, 40, 50.

EARTH

This is a multivariate adaptive regression splines algorithm which accounts for interactions and non-linear relationships using splines. Standard setting were used with no additional tuning.

HAL

The highly adaptive lasso accounts for interactions and non-linear relationships using basis functions. The hyperparameters were: interaction levels = 2, number of knots = 10 and 5, reduce basis = 0.1, performance measure = deviance, cross validation folds stratified by outcome, and outcome type = binomial. For tuning, The lambda parameter was tuned using a grid of 100 values, CV select minimum lambda. The maximum interaction degrees were tested at 2, 3. The smoothness orders were tuned between 1 and 2. The number of knots for basis terms and interaction terms were tuned for (25,10), (10,5), (5,2). The reduce basis parameter was tuned between 0.1 and 0.2.

SuperLearner

Ensebmle meta learner, uses stacking to combine the above algorithms to achieve better performance. The parameters for the SuperLearner were set at: cross validation folds = 3, CV folds stratified by the outcome, Cluster id = PICUs, outcome type = binomial, metalearners = solnp, logistic-binomial, and loss-loglik-binomial.

5. **Sensitivity analysis**

Multiple imputations: To assess the impact of missing data on the estimate of the ATT, we performed multiple imputations to reincorporate data from the missing records, then re-estimated the ATT on the multiply imputed datasets to assess if and how the ATT estimate was biased as a result of the missing data, and what is the magnitude or direction of this bias. There were 9 variables with <99% complete values out of 26. We imputed the values for each missing variable conditional on the set of complete variables. The completeness rate for the variables with missing data were IMD Decile (98.2%), Maternal multiple births, e.g. twin births (97.5%), Congenital anomaly (95.9%), Chronic complex conditions (92.8%), Prematurity condition (91.4%), Perinatal infection (91.2%), Birth weight (90.2%) , Number of maternal diagnoses derived from HES ICD10 codes (89.0%), and Born by C-section (88.3%). Missing data for the variables were imputed using multiple imputations by chained equations (MICE). MICE relies on the assumption that the data are missing at random, meaning that the probability of a value is missing depends only on the observed values of relevant variables. The MICE procedure involves a series of regression models where each incomplete variable is modelled conditional on other variables in the data. The R package **`mice`** was used for this purpose. Due to the size of the dataset and in the interest of time, five imputations were carried out with 5 initial iterations. The R package **`howManyImputations`** was used to assess if choosing five imputations was a reasonable limit for this dataset.

Diagnostics for the imputed data were carried out to assess the distribution of the imputed data relative to the complete cases data. G-computation and AIPW estimation were performed on the imputed data, all using logistic regression for the propensity score model and the outcome model with no spline terms for birth weight. The estimates obtained in each imputed set were then pooled using Rubin's rules. The parametric g-computation was performed using the R package **`marginaleffects`**. For the missing covariates, they were assumed to be missing at random (MAR) conditional on the fully observed covariates, the outcome, and the exposure. Since the exposure and outcome were fully observed, no assumptions were made for these variables.

Table S1. Variables missingness rates

Variable	Complete %
Death on first admission to PICU	100.0
Female sex	100.0
Other diagnoses in PICU, categorised	100.0
Ethnicity	100.0
Indicator of health at birth	100.0
Recorded comorbidities in PICU, categorised	100.0
Emergency admissions pre-PICU	100.0
Source of admission to hospital	100.0
Year of admission to PICU	100.0
Risk factors during pregnancy or delivery	100.0
Episodes of child admission to hospital, pre PICU	100.0
PICU admission age in months	100.0
Paediatric index of mortality	100.0
Days in hospital	100.0
Admission to PICU from the same hospital	100.0
Planned versus unplanned admission to PICU	99.9
Primary diagnostic group in PICU	99.7
Maternal age in years	99.2
Preterm birth (<37 weeks gestation)	99.1
IMD Decile	98.2
Maternal multiple births, e.g. twin births	97.5
Congenital anomaly*	95.9
Chronic complex conditions	92.8
Prematurity condition	91.4
Perinatal infection	91.2
Birth weight	90.2
Number of maternal diagnoses**	89.0
Born by C-section	88.3

*See section 2

**Derived from HES ICD10 codes

6. **Comparison of analysis cohort with full dataset**

Table S2. Comparisons of full PICU dataset, linked dataset, study cohort, and the complete records dataset

Characteristic	All PICU N = 101,919[1]	Linked records N =66,779[1]	Study cohort N = 64,495[1]	Study cohort complete records N = 48,500[1]
**Sex**				
Female	43649 (42.8%)	28178 (42.2%)	27236 (42.2%)	20412 (42.1%)
Male	58270 (57.2%)	38601 (57.8%)	37259 (57.8%)	28088 (57.9%)
**Ethnicity group**				
Asian	11988 (11.8%)	8966 (13.4%)	8739 (13.5%)	6366 (13.1%)
Black	9281 (9.1%)	4203 (6.3%)	4076 (6.3%)	2946 (6.1%)
Mixed	3982 (3.9%)	2892 (4.3%)	2819 (4.4%)	2111 (4.4%)
Other	3554 (3.5%)	2059 (3.1%)	1990 (3.1%)	1354 (2.8%)
White	73114 (71.7%)	48659 (72.9%)	46871 (72.7%)	35723 (73.7%)
Missing	0	0	0	0
**Primary diagnostic group**				
Cardiovascular	29820 (29.3%)	20533 (30.8%)	19963 (31.0%)	14911 (30.7%)
Endocrine / metabolic	2643 (2.6%)	1553 (2.3%)	1499 (2.3%)	1135 (2.3%)
Gastrointestinal	6378 (6.3%)	4292 (6.4%)	4197 (6.5%)	3108 (6.4%)
Infection	5864 (5.8%)	4010 (6.0%)	3836 (6.0%)	2904 (6.0%)
Musculoskeletal	3535 (3.5%)	1018 (1.5%)	1005 (1.6%)	733 (1.5%)
Neurological	11181 (11.0%)	7200 (10.8%)	6843 (10.6%)	5228 (10.8%)
Oncology	3459 (3.4%)	1952 (2.9%)	1912 (3.0%)	1408 (2.9%)
Other	7492 (7.4%)	4516 (6.8%)	4387 (6.8%)	3325 (6.9%)
Respiratory	28655 (28.2%)	20190 (30.3%)	19387 (30.1%)	14739 (30.4%)
Trauma	2648 (2.6%)	1334 (2.0%)	1291 (2.0%)	1009 (2.1%)
Missing	244	181	175	0
**Year of admission into PICU**				
2010	9979 (9.8%)	7014 (10.5%)	6659 (10.3%)	4985 (10.3%)
2011	9149 (9.0%)	6735 (10.1%)	6494 (10.1%)	5016 (10.3%)
2012	9467 (9.3%)	7007 (10.5%)	6693 (10.4%)	5208 (10.7%)
2013	9508 (9.3%)	6745 (10.1%)	6532 (10.1%)	5106 (10.5%)
2014	9522 (9.3%)	6558 (9.8%)	6330 (9.8%)	4902 (10.1%)
2015	9942 (9.8%)	6623 (9.9%)	6414 (9.9%)	4958 (10.2%)
2016	11266 (11.1%)	6716 (10.1%)	6538 (10.1%)	4957 (10.2%)
2017	10862 (10.7%)	6454 (9.7%)	6272 (9.7%)	4625 (9.5%)
2018	11070 (10.9%)	6457 (9.7%)	6270 (9.7%)	4548 (9.4%)
2019	11154 (10.9%)	6470 (9.7%)	6293 (9.8%)	4195 (8.6%)
**Decile of deprivation score***				
0**	1983 (2.2%)	430 (0.7%)	414 (0.7%)	228 (0.5%)
1 (Most deprived)	14219 (16.0%)	11128 (17.0%)	10822 (17.1%)	8681 (17.9%)
2	12023 (13.5%)	9195 (14.0%)	8899 (14.1%)	6938 (14.3%)
3	10714 (12.1%)	8047 (12.3%)	7796 (12.3%)	5957 (12.3%)
4	9072 (10.2%)	6872 (10.5%)	6626 (10.5%)	4998 (10.3%)

Characteristic	All PICU<br>N = 101,919[1]	Linked records<br>N =66,779[1]	Study cohort<br>N = 64,495[1]	Study cohort complete records<br>N = 48,500[1]
5	7958 (9.0%)	5933 (9.0%)	5673 (9.0%)	4274 (8.8%)
6	7514 (8.5%)	5553 (8.5%)	5330 (8.4%)	4051 (8.4%)
7	6637 (7.5%)	4954 (7.6%)	4786 (7.6%)	3607 (7.4%)
8	6518 (7.3%)	4783 (7.3%)	4619 (7.3%)	3530 (7.3%)
9	6255 (7.0%)	4447 (6.8%)	4274 (6.7%)	3182 (6.6%)
10	5859 (6.6%)	4227 (6.4%)	4084 (6.4%)	3054 (6.3%)
Missing	13,167	1,210	1,172	0
**Other PICU diagnoses, categorised**				
0	55371 (54.3%)	35087 (52.5%)	34097 (52.9%)	25778 (53.2%)
1	24742 (24.3%)	16531 (24.8%)	15866 (24.6%)	11976 (24.7%)
2	11059 (10.9%)	7658 (11.5%)	7310 (11.3%)	5458 (11.3%)
3+	10747 (10.5%)	7503 (11.2%)	7222 (11.2%)	5288 (10.9%)
**Planned/unplanned admission to PICU**				
No	38003 (37.3%)	22758 (34.1%)	22253 (34.5%)	16663 (34.4%)
Yes	63809 (62.6%)	43954 (65.8%)	42179 (65.4%)	31837 (65.6%)
	107	67	63	0
**Indicator of health at birth**				
No	74001 (83.0%)	55374 (82.9%)	53539 (83.0%)	40080 (82.6%)
Yes	15172 (17.0%)	11405 (17.1%)	10956 (17.0%)	8420 (17.4%)
Missing	12,746	0	0	0
**Preterm birth (<37 weeks gestation)**				
No	64635 (76.8%)	45546 (76.5%)	44015 (76.4%)	33848 (69.8%)
Yes	19575 (23.2%)	14029 (23.5%)	13593 (23.6%)	14652 (30.2%)
Missing	17,709	7,204	6,887	0
**PICU admission from same hospital**				
No	38149 (37.5%)	27328 (40.9%)	25666 (39.8%)	18904 (39.0%)
Yes	63714 (62.5%)	39417 (59.1%)	38797 (60.2%)	29596 (61.0%)
Missing	56	34	32	0
**Emergency admissions pre-PICU**				
Elective	9741 (10.9%)	5871 (8.8%)	5733 (8.9%)	4220 (8.7%)
Emergency	79432 (89.1%)	60908 (91.2%)	58762 (91.1%)	44280 (91.3%)
Missing	56	34	32	0
**Born by C-section**				
No	44652 (66.3%)	38979 (66.1%)	37649 (66.1%)	31802 (65.6%)
Yes	22678 (33.7%)	19983 (33.9%)	19321 (33.9%)	16698 (34.4%)
Missing	34,589	7,817	7,525	0
**Risk factors during pregnancy/delivery*** **				
No	91908 (90.2%)	57656 (86.3%)	56160 (87.1%)	41093 (84.7%)
Yes	10011 (9.8%)	9123 (13.7%)	8335 (12.9%)	7407 (15.3%)
**Source of admission to hospital**				

Characteristic	All PICU<br>N = 101,919[1]	Linked records<br>N =66,779[1]	Study cohort<br>N = 64,495[1]	Study cohort complete records<br>N = 48,500[1]
Medical facility	19293 (21.7%)	14499 (21.8%)	13865 (21.6%)	10219 (21.1%)
Non-medical facility	69499 (78.3%)	52020 (78.2%)	50375 (78.4%)	38281 (78.9%)
Missing	13,127	260	255	0
**Multiple births**				
No	56714 (94.9%)	53225 (94.7%)	51446 (94.7%)	46057 (95.0%)
Yes	3017 (5.1%)	2965 (5.3%)	2876 (5.3%)	2443 (5.0%)
Missing	42188	10,589	10,173	0
**PICU comorbidities, categorised**				
0	70233 (68.9%)	47663 (71.4%)	46060 (71.4%)	34655 (71.5%)
1	15787 (15.5%)	9899 (14.8%)	9524 (14.8%)	7179 (14.8%)
2	6952 (6.8%)	4161 (6.2%)	4012 (6.2%)	3009 (6.2%)
3+	8947 (8.8%)	5056 (7.6%)	4899 (7.6%)	3657 (7.5%)
**Chronic complex conditions**				
No	36460 (48.3%)	34249 (55.2%)	33187 (55.5%)	28206 (58.2%)
Yes	38969 (51.7%)	27744 (44.8%)	26640 (44.5%)	20294 (41.8%)
Missing	26,490	4,786	4,668	0
**Perinatal infection**				
No	50704 (72.9%)	45956 (75.4%)	44392 (75.5%)	37775 (77.9%)
Yes	18838 (27.1%)	14969 (24.6%)	14426 (24.5%)	10725 (22.1%)
Missing	32,377	5,854	5,677	0
**Congenital anomaly**				
No	24357 (30.7%)	21515 (33.6%)	20657 (33.4%)	17559 (36.2%)
Yes	54947 (69.3%)	42550 (66.4%)	41223 (66.6%)	30941 (63.8%)
Missing	22,615	2,714	2,615	0
**Maternal substance related risk factors**				
No	88679 (81.0%)	66398 (99.4%)	64126 (99.4%)	48217 (99.4%)
Yes	20763 (19.0%)	381 (0.6%)	369 (0.6%)	283 (0.6%)
Missing	12,746	0	0	0
**Prematurity condition**				
No	49289 (70.4%)	44289 (72.5%)	42746 (72.5%)	36501 (75.3%)
Yes	20763 (29.6%)	16800 (27.5%)	16232 (27.5%)	11999 (24.7%)
Missing	31,867	5,690	5,517	0
**PIM version 3 (risk of death)**	0.02 (0.01, 0.04)	0.02 (0.01, 0.04)	0.02 (0.01, 0.04)	0.02 (0.01, 0.04)
**Birth weight (kg)**	3.3 (2.7, 3.9)	3.1 (2.6, 3.5)	3.1 (2.5, 3.5)	3.1 (22.5, 3.5)
Missing	20080	6,605	6,348	0
**Number of admissions to PICU**	1 (1, 1)	1 (1, 2)	1 (1, 2)	1 (1, 2)
**Gestational age at birth, in weeks**	39.0 (37.0, 40.0)	38.0 (37.0, 40.0)	38.0 (37.0, 40.0)	38 (35, 40)
Missing	17709	7,204	6,887	0
**PICU admission age in months**	10 (2, 48)	6 (1, 26)	6 (1, 26)	6 (1, 25)
**Maternal age in years**	29 (24, 34)	29 (24, 34)	29 (24, 34)	29 (24, 34)
Missing	26817	548	531	

Characteristic	All PICU<br>N = 101,919[1]	Linked records<br>N =66,779[1]	Study cohort<br>N = 64,495[1]	Study cohort complete records<br>N = 48,500[1]
**Number of maternal diagnoses**	6 (3, 12)	7 (3, 12)	7 (3, 12)	7 (3, 12)
Missing	37116	7,330	7,112	
**Length of PICU stay (days)**	2.2 (1.0, 5.0)	2.5 (1.0, 5.1)	2.4 (1.0, 5.1)	2.4 (1.0, 5.1)

[1]n (%); Median (IQR). Study cohort: Records for all children age 0-8 years, and linked to both child and maternal HES records. Study cohort: Records for all children age 0-8 years, and linked to both child and maternal HES records. * The deprivation score used was the Index of Multiple Deprivation 2015. ** A deprivation decile of 0 was for children with an English birth record in HES, but resident in Wales, and had an admission to an English PICU. *** Refer to section 2 for ICD10 code list.

7. Additional descriptive statistics

Paediatric Index of Mortality (PIM)

We carried out a comparison of the mean, median and 10th to 90th centiles of the PIM version 3 score between male and female children. PIM values closer to 1 indicate higher risk of death. Both males and females show very similar risk of death on presentation to PICU as measured by the PIM score.

The majority of children presenting to PICU (106,471/114,357, 93%) had a risk of death (on a scale from 0 to 1) within the 10th centile derived from the PIM score. Within the 10th centile of risk of death, 60% of all deaths occurred (3731/6220 deaths), and female and male mortality rates were 3.69% and 3.37% respectively (female to male ratio = 1.10). The group of children in the remaining centiles (N = 7,886) contributed 40% (2489/6220 deaths) of the overall deaths in PICU with female and male death rates being 32.65% and 30.81% respectively (female to male ratio = 1.06).

Table S3. Mean and median sex-specific risk of death, and by centiles of PIM score.

	**Female**, N = 49,612	**Male**, N = 64,745
PIM3 2019 Recalibration		
Mean	0.04	0.04
Median	0.02	0.02
10%	0.00	0.00
20%	0.01	0.01
30%	0.01	0.01
40%	0.01	0.02
50%	0.02	0.02
60%	0.03	0.03
70%	0.04	0.04
80%	0.05	0.05
90%	0.08	0.08

PIM3 2019 recalibration: Paediatric Index of Mortality version 3, recalibrated in 2019

Primary diagnostic groups

Although the individual diagnosis codes for each child are recorded in PICANet, these diagnoses are also grouped into 11 primary diagnostic categories. The category with the largest number of children is Cardiovascular (N = 31,935, 28%), followed by Respiratory (N = 30,294, 26%), then Neurological (12,722, 11%). There were some differences in the distributions of admissions and deaths within each sex. The percentages of males and females for diagnostic group musculoskeletal show the greatest difference; females were 59% of the group total(from the total admissions and not as shown in Table S4).

Overall, the female to male ratio of deaths ranged from 0.8 (endocrine/metabolic) to 1.56 (trauma). The percentage of respiratory diagnoses was higher in males than females(28.8% females, 30.7% males), but the risk of deaths was higher for females than males, (3.9% females, 2.8% males, risk ratio = 1.39). The percentage of musculoskeletal diagnoses was higher for females than males, but a lower risk of death compared to males, RR = 0.63. It appears that where the prevalence of a diagnostic group is lower, the risk of death is higher, and vice versa.

Table S4. Distributions of sex-specific admissions and deaths in PICU by primary diagnostic groups for children age 0-17 years admitted to PICU between 2010 and 2019

	**Within sex Percent admissions**		**Within diagnostic groups Percent deaths**		**Death in PICU***
**Primary diagnostic group**	**Female %**	**Male %**	**Female %**	**Male %**	**F/M RR**
Cardiovascular	28.41	27.57	6.82	6.44	1.06
Respiratory	25.14	27.55	4.14	3.13	1.32
Neurological	11.13	11.13	6.63	6.32	1.05
Other	7.35	7.81	5.68	6.07	0.93
Musculoskeletal	7.27	3.87	0.55	1.56	0.36
Gastrointestinal	5.74	6.37	6.28	5.09	1.23
Infection	5.46	5.61	7.90	7.57	1.04
Oncology	3.83	3.50	6.05	6.62	0.91
Endocrine / metabolic	3.03	2.64	9.77	11.97	0.82
Trauma	2.37	3.72	4.41	3.61	1.22
Missing	0.25	0.23	4.92	0.66	7.43
Total	100.00	100.00			

F/M RR: Crude female to male risk ratio
*These are all deaths that occurred during the first and subsequent admissions to PICU for the duration of the study period

The same breakdown, for ages 0 to 8 years of mortality by diagnostic grouping is presented in Table S5.

Table S5. Distributions of sex-specific admissions and deaths in PICU by primary diagnostic groups for children age 0 to 8 years and admitted to PICU between 2010 and 2019

	**Within sex Percent admissions**		**Within diagnostic groups Percent deaths**		**Death in PICU***
**Primary diagnostic group**	**Female %**	**Male %**	**Female %**	**Male %**	**F/M RR**
Cardiovascular	32.12	29.37	7.07	6.75	1.05
Respiratory	28.79	30.69	3.94	2.84	1.39
Neurological	10.87	10.38	6.04	5.91	1.02
Other	6.68	7.44	6.52	6.56	0.99
Gastrointestinal	6.19	6.62	6.71	5.38	1.25
Infection	5.91	6.00	7.73	7.49	1.03
Oncology	3.22	2.87	6.28	6.76	0.93
Endocrine / metabolic	2.41	2.26	11.17	13.94	0.80
Trauma	1.82	2.35	5.43	3.48	1.56
Musculoskeletal	1.74	1.78	2.53	2.57	0.98
Missing	0.26	0.23	6.00	0.81	7.38
Total	100.00	100.00			

F/M RR: Female to male risk ratio.
*These are all deaths that occurred during the first and subsequent admissions to PICU for the duration of the study period

8. Visual abstract co-authored by a group of young persons

Sex differences in Paediatric Intensive Care Mortality

Calista Stalker-Firth[1], Jasmine Stalker-Firth[1], Muhtasim Abrar[2], Mahir Daiyan[1], Ofran Almossawi[3,4]

(1) Secondary school student, (2) University undergraduate, (3) Great Ormond Street Hospital, (4) UCL Institute of Child Health

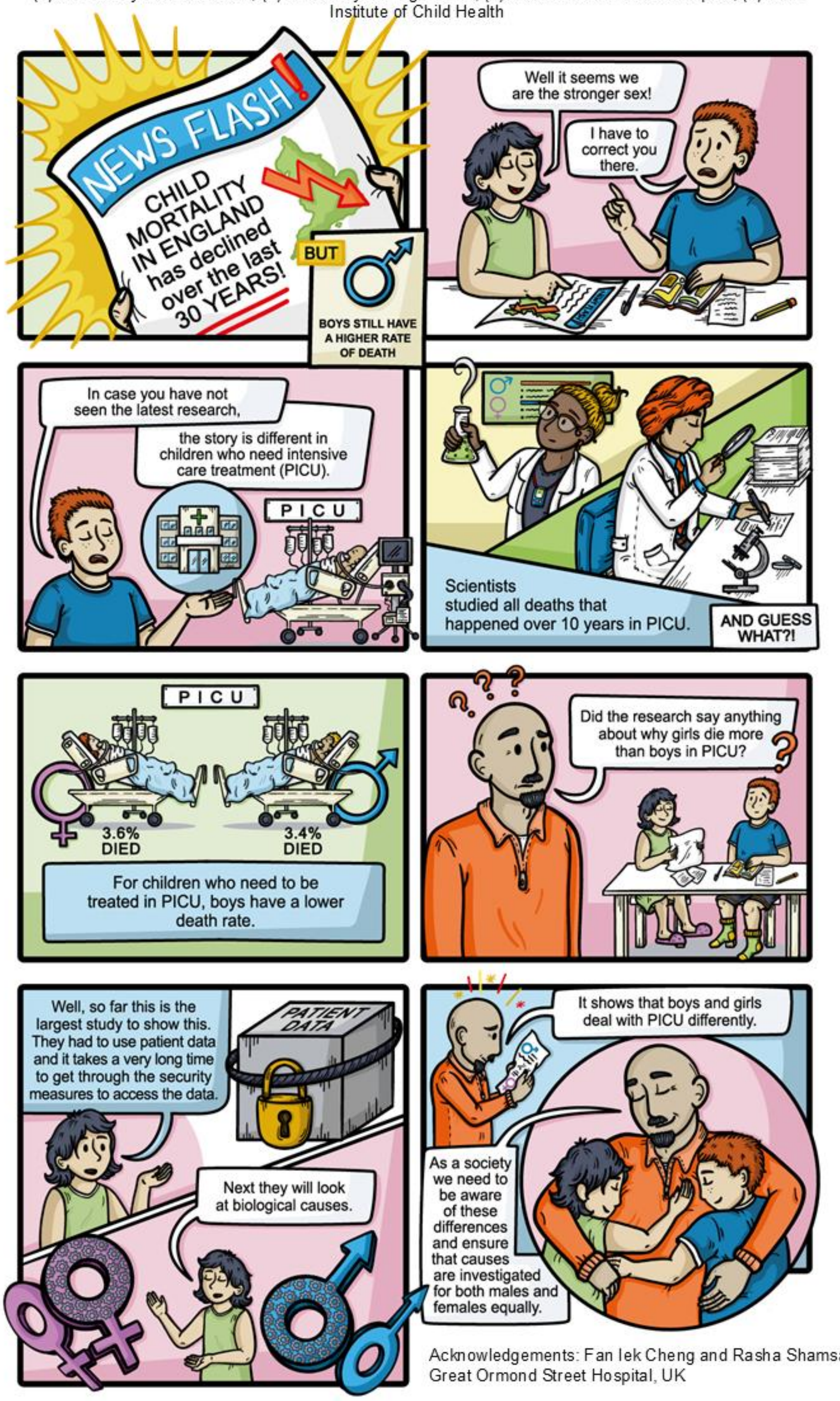

Acknowledgements: Fan Iek Cheng and Rasha Shamsa, Great Ormond Street Hospital, UK

*PICU= Paediatric Intensive Care Unit

inkythinking.com

Research funded by National Institute for Health and Care Research
